# Pectoralis Major Rupture: A Case Report

**DOI:** 10.7759/cureus.29512

**Published:** 2022-09-23

**Authors:** Kristo Qylafi, Yousif Alkhalfan

**Affiliations:** 1 Orthopaedics and Trauma, St Thomas' Hospital, London, GBR

**Keywords:** trauma of pectoralis major, pectoralis major injury, pectoralis major rupture, rupture of sternal head of pectoralis major, isolated injury of pectoralis major

## Abstract

Pectoralis major (PM) ruptures are relatively rare injuries occurring mostly in men 20 to 40 years of age. Weightlifting or bench pressing is the most common mechanism of injury. Although an uncommon injury, a high degree of clinical suspicion should be maintained since early surgical treatment has been shown to be beneficial. We present a case of a 51-year-old male with no known past medical history, who presented to the emergency department with sudden onset right chest pain after bench pressing (approximately 180kg). Physical examination revealed PM rupture with ecchymosis and loss of shoulder contour, as well as bulking over the right chest. He was otherwise neurovascularly intact. The right shoulder x-ray showed no fracture, dislocation or other bony abnormality. The diagnosis was confirmed by an urgent MRI scan which revealed a complete rupture of the sternal head of the PM, and the patient underwent right PM tendon repair nine days after the injury.

## Introduction

Pectoralis major (PM) ruptures are uncommon injuries [1]. PM provides arm adduction and internal rotation, while it also provides some flexion [2-4]. Injuries of the PM mostly occur in young males during weightlifting or bench pressing [1-3]. Literature supports that surgical treatment is advantageous for patients [1,5]. Although the condition is well known among orthopedic surgeons, due to its rarity, a high degree of clinical suspicion is required for the diagnosis not to be missed. We report a case of PM rupture in a 51-year-old man who presented to our hospital after bench pressing.

## Case presentation

**Figure 1:**
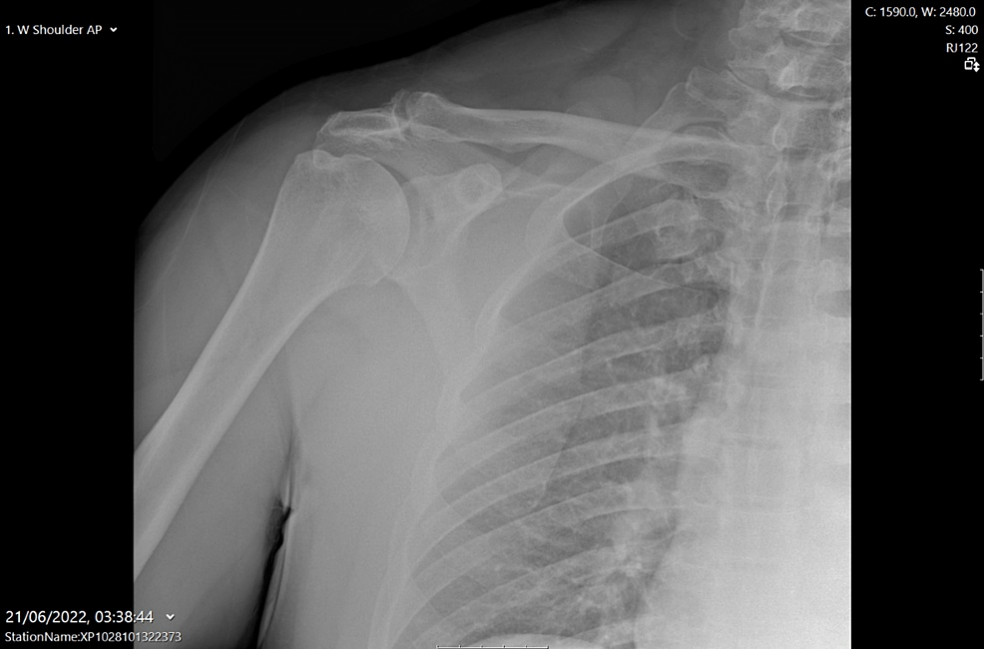
Right shoulder x-ray No bony abnormalities are evident

**Figure 2:**
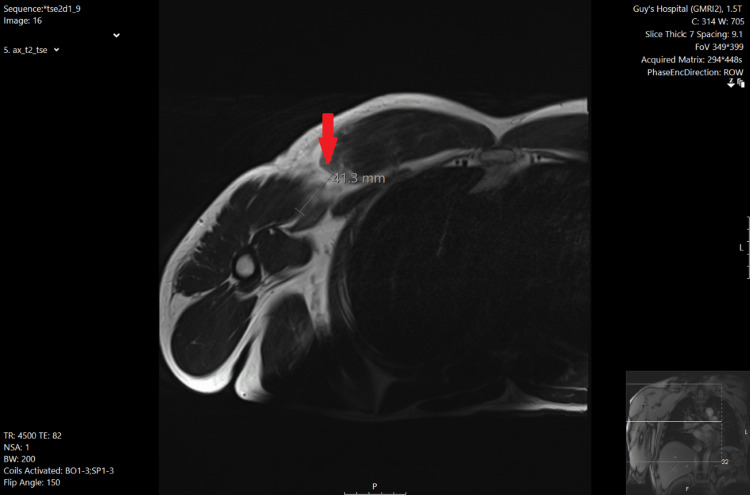
Right pectoralis major MRI scan Arrow demonstrating the PM rupture Distance between ruptured free edges (retraction) is measured 41.3mm

A 51-year-old male presented to the emergency department after experiencing sudden pain in his right chest, accompanied by a tearing sensation while bench pressing (approximately 180kg). He was a construction worker who exercised with a lot of weight training and denied any steroid use, although he admitted to having used steroids in the past. His past medical history was unremarkable, and he was a non-smoker. On examination, he had evidence of PM rupture with ecchymosis and loss of shoulder contour, as well as bulking over the right chest. The shoulder range of movement was preserved, although decreased muscle power in arm adduction and internal rotation was noticed. The patient had a Medical Research Council (MRC) scale of 3 out of 5 in shoulder adduction (Active movement against gravity) and an MRC scale of 4 out of 5 in shoulder internal rotation (Active movement against resistance), non-pain related. He was otherwise neurovascularly intact throughout his right upper limb. Plain radiographs of the right shoulder were obtained (Figure 1) which showed no fracture, dislocation or other bony abnormality. Two days after an urgent magnetic resonance imaging (MRI) scan was performed (Figure 2), which revealed a PM rupture with retraction and the patient underwent right PM tendon repair nine days after the injury. Specifically, a deltopectoral approach was used and the two origin heads of the PM were shown to be intact. A rupture in the musculotendinous junction was recognized and subsequently, direct sutures were applied.

## Discussion

Epidemiology

The vast majority of the cases occur in young men between 20 and 40 years [1-3]. Rupture occurs mainly during bench pressing [2,5] and weigh-lifting accounts for nearly 50% of the cases [3]. However, other activities such as rugby or boxing have been identified [2]. The presented case was outside the expected age range, which highlights the fact that PM should be considered in older individuals presenting with a consistent mechanism of injury.

Anatomy/classification

PM is composed of two heads: The clavicular head originates from the medial half of the clavicle and it is shorter. The sternal head originates from the second to sixth ribs, the costal margin of the sternum, and the aponeurosis of the external oblique. The two heads fuse to a broad tendon at the intertubercular sulcus [3]. Specifically, they both insert through an anterior and posterior layer at the lateral lip of the bicipital groove [3]. PM's main functions are adduction and internal rotation of the arm, while it also provides some flexion [2,3,4]. The traditional classification of Tietjen divides PM injuries into three groups: contusion, partial tear, and complete tear [2-4]. Furthermore, each injury is subdivided by anatomical location to the muscle belly, the musculotendinous junction (24%-29%), and tendinous insertion (59%-65%) [2,3].

**Table 1:** Tietjen classification of PM ruptures

Type	Description
I	Sprain/Contusion
II	Partial Tear
IIIA	Complete Tear - Sternoclavicular Origin
IIIB	Complete Tear - Muscle Belly
IIIC	Complete Tear - Musculotendinous Junction
IIID	Complete Tear – Tendon Insertion

Pathogenesis/etiology

PM tears occur during excessive tension or, less commonly, after a direct trauma [6]. Specifically, rupture occurs when tension is applied to an eccentrically contracting muscle [3,4]. This is the case of maximal contraction when the arm is externally rotated, extended, and abducted [4]. Less commonly, rupture may result from a direct blow [4,6]. Muscle structure allows for maximum muscle power production but has also as consequence disproportionately high fiber excursion in the inferior sternocostal head. This phenomenon is believed to be the explanation behind PM rupture during mechanical stress in the disadvantageous position (e.g., bench press) [3], which was also the mechanism of PM rupture in the presented case.

Presentation

Classic history includes sudden onset of pain, accompanied by a “pop” sensation [2,7]. On examination, there is usually bruising and swelling of the affected anterolateral chest wall [2,3]. Tenderness over the humeral insertion is common [3,7]. Loss of the axillary fold can be seen [2,3,7], although this finding may be obscured by the tissue swelling [2,7]. Resisted adduction is helpful in testing strength as muscle power may be decreased [2,7]. Full-thickness tears have a characteristic “gap.” However, the axillary fold and not the fascial sheath should be palpated [3]. On examination, our patient had evidence of PM rupture with ecchymosis over the right shoulder area and loss of shoulder contour, as well as bulking over the right chest. Although no gap could be palpated and shoulder range of movement was preserved, muscle power in arm adduction and internal rotation was significantly decreased (MRC scale 3 out of 5 in shoulder adduction and 4 out of 5 in shoulder internal rotation).

Diagnosis

Initial imaging investigation should include plain radiographs to exclude concomitant bone injuries [4,8]. Pain radiographs may be useful only in rare cases of bone avulsion [2-4]. This occurs in 2%-5% of the cases [3,8]. U/S is an adjunct when MRI cannot be performed [2]. Its usefulness relies on the fact that it is a low-cost and available imaging modality [9]. However, U/S is an operator-dependent modality and is shown to produce false negative results [3,9]. MRI is the investigation of choice [2,10]. It can distinguish between partial and full thickness tears [10] thus helping with the surgical planning [2,10]. In our case, 48 hours after MRI revealed an intramuscular complete rupture of the sternal head of the PM.

Treatment

Literature supports by far surgical management [2,5]. Repair techniques vary considerably but usually consist of transosseous fixation, suture fixation, anchor fixation, and cortical button fixation. For chronic ruptures, reconstruction with the use of autografts or allografts is described [2,3,11]. Specific repair technique depends on the site of rupture: Musculotendinous junction injuries are repaired by direct suturing, while avulsion injuries are anatomically reduced and internally fixated [4]. Although no guidelines regarding optimal surgical timing exist, literature generally shows that acute repairs are easier and lead to improved results [3,12]. In chronic settings, adhesions between ruptured muscle and chest wall may complicate the procedure [4]. Conservative management is reserved for the elderly population or individuals who do not wish or are not medically fit for surgery [2,3]. The protocol involves sling immobilization with immediate passive exercises and unrestrictive activity allowed after two to three months [2,3]. Contact sports should only be initiated after 5-6 months [3]. However, recent studies show that the power of adduction and internal rotation is permanently diminished when conservative management is chosen [4,13]. In our case, the patient underwent right PM tendon repair nine days after the injury. On follow-up in the second and sixth post-operative week, no postoperative complication was evident, and the patient was referred for physiotherapy. Currently, the patient demonstrates almost normal muscle power (MRC scale 5 out of 5) in all shoulder movements.

## Conclusions

PM ruptures are uncommon injuries and commonly occur in young men between 20 and 40 years. Patients usually present with shoulder pain and weakness after a strenuous activity and a diagnosis can be made with MRI. Surgical treatment is considered the optimal treatment with conservative management reserved for elderly population or individuals who do not wish to undergo an operation.
